# Applications of Nanobiotechnology in Medicine

**DOI:** 10.3390/life16020302

**Published:** 2026-02-10

**Authors:** David Aebisher, Klaudia Dynarowicz, Izabela Rudy, Kacper Rogóż, Dorota Bartusik-Aebisher, Aleksandra Kawczyk-Krupka

**Affiliations:** 1 Department of Photomedicine and Physical Chemistry, Faculty of Medicine, University of Rzeszów, 35-310 Rzeszów, Poland; 2 Department of Biochemistry and General Chemistry, Faculty of Medicine, University of Rzeszów, 35-310 Rzeszów, Poland; kdynarowicz@ur.edu.pl; 3 Student Scientific Club of Biochemists URCell, Faculty of Medicine, University of Rzeszów, 35-310 Rzeszów, Poland; 4 Department of Internal Diseases, Angiology and Physical Medicine, Center for Laser Diagnostics and Therapy, Faculty of Medical Sciences in Zabrze, Medical University of Silesia, 40-055 Katowice, Poland

**Keywords:** nanobiotechnology, drug delivery, targeted therapy, diagnostics, tissue engineering, immunotherapy, theranostics

## Abstract

Nanobiotechnology, defined as the application of nanotechnology in biology and medicine, refers to the use of nanometric structures to solve complex clinical problems through precise interaction at the molecular level. Nanostructures such as lipid, polymer, and metallic nanoparticles offer unique properties that enable improved therapeutic and diagnostic efficacy and the integration of diagnostic and therapeutic functions within the concept of theranostics. Major applications of nanobiotechnology include targeted drug delivery in cancer, infection, and gene therapy; advanced molecular diagnostics and biosensors; tissue engineering and regeneration; and immune system modulation through modern nanotechnology-based vaccines and immunotherapies. The clinical significance of these technologies lies in their ability to improve drug bioavailability, minimize adverse effects, increase sensitivity in early disease detection, and support personalized treatment strategies. Nanobiotechnology also contributes to the development of precision medicine by combining diagnostics and therapy within a single nanosystem. Despite promising results, significant challenges remain related to safety, biocompatibility, toxicity, and translation from laboratory studies to clinical applications. Further research is needed to standardize methods, assess the long-term health impact of nanomaterials, and develop regulatory frameworks to fully realize the potential of nanobiotechnology in medicine.

## 1. Introduction

Nanobiotechnology is an interdisciplinary field of science that combines the principles of nanotechnology with biology and medicine, aiming to utilize structures and materials in the nanometer range (1–100 nm) to address biological and clinical challenges. In the medical context, this field is referred to as nanomedicine and encompasses both diagnostic and therapeutic applications. Nanobiotechnology forms the foundation of modern approaches to drug delivery, the development of nanosensors, and the design of nanostructures that enhance treatment efficacy and disease diagnosis [1,2]. In medicine, there is a growing need to address challenges that traditional technologies cannot effectively resolve, such as precise drug delivery to specific tissues or the early detection of disease biomarkers. Nanostructures enable interactions at the molecular level, leading to improved therapeutic bioavailability, reduced side effects, and increased sensitivity of diagnostic methods. Due to their unique physicochemical properties, nanosystems can cross biological barriers, such as the blood–brain barrier, thereby opening new therapeutic possibilities [3,4]. Research on nanobiotechnology in medicine has developed rapidly over the past two decades. Initially, efforts focused on understanding the fundamental properties of nanostructures and exploring their potential applications. More recent studies have shifted toward specific clinical applications, including nanocarriers for cancer therapy, diagnostic nanosensors, and therapeutic nanosystems. Numerous scientific reviews and experimental studies document the growing importance of nanobiotechnology in enabling personalized therapies and more effective medical procedures while also highlighting the challenges associated with translating laboratory research into clinical practice [5,6,7]. Despite the rapidly growing number of review articles addressing individual aspects of nanobiotechnology, such as nanoparticle-based drug delivery systems, nanodiagnostics, immunotherapy or tissue engineering, many of these reports focus on narrowly defined applications or remain largely descriptive. In recent years, increasing attention has been drawn to the discrepancy between promising preclinical results and the limited number of nanobiotechnological solutions that successfully reach clinical implementation. Moreover, challenges related to biological complexity, safety, regulatory pathways and large-scale manufacturing are often discussed separately, without an integrated translational perspective. Unlike previously published reviews, this work offers a comprehensive and integrated overview of nanobiotechnology in the main therapeutic and diagnostic fields. The originality of this work lies in its comparative approach, in which different classes of nanomaterials are evaluated for analytical performance, therapeutic efficacy, and translational potential. Furthermore, this review considers the latest developments from 2024 to 2025, with a particular emphasis on hybrid nanomaterials, theranostic systems, and clinically relevant design issues, thus offering updated and structured knowledge with practical medical applications.

## 2. Nanomaterials Used in Medicine

### 2.1. Organic, Inorganic, and Hybrid Nanomaterials in Medicine

#### 2.1.1. Liposomes and Lipid Nanoparticles

**Figure 1:**
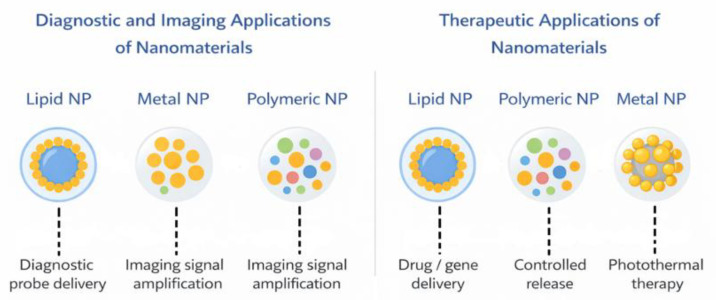
Diagnostic, imaging, and therapeutic applications of nanomaterials in medicine. Lipid, polymeric, and metallic nanoparticles are utilized as platforms for diagnostic probe delivery and imaging signal amplification, as well as for therapeutic purposes including drug and gene delivery, controlled release of active compounds, and photothermal therapy.

Liposomes are spherical nanostructures composed of a lipid bilayer that can encapsulate both water-soluble and hydrophobic substances. Due to their structure, liposomes improve drug stability, protect therapeutic agents from degradation, and enable controlled release at the site of action. They are widely studied as carriers for anticancer and immunotherapeutic drugs (Figure 1), and their surface modifications, such as polyethylene glycol (PEG) coating, can prolong blood circulation time and improve therapeutic bioavailability [8,9] (Table 1).

#### 2.1.2. Polymeric Nanoparticles and Dendrimers

Polymeric nanoparticles are made from natural or synthetic polymers that are biocompatible and biodegradable. Due to their ability to modify their surface and provide the controlled release of pharmaceuticals (Figure 1), they are used as carriers for drugs, peptides, and nucleic acids. Dendrimers are branched macromolecules with a precisely controlled structure that, thanks to the large number of functional groups on their surface, can be functionalized and bind to various therapeutic molecules. Both classes of nanomaterials increase drug delivery efficiency and bioavailability, which is particularly important in targeted therapy [8,10].

#### 2.1.3. Metallic and Semiconducting Nanoparticles

Metallic nanoparticles, such as those based on gold or iron, possess unique optical and magnetic properties that are used in both therapy and diagnostics (Figure 1). Gold nanoparticles can serve as contrast agents in imaging or as carriers for anticancer drugs, while magnetic iron nanoparticles enable magnetically guided drug delivery and their use in magnetic resonance imaging (MRI). Semiconducting nanoparticles, such as quantum dots, are being investigated for bioimaging and biosensing applications due to their fluorescent properties, which enable the tracking of biological processes in vitro and in vivo [8,11].

#### 2.1.4. Hybrid Nanobiological Systems

Hybrid nanomaterials combine organic and inorganic components, combining the advantages of different classes of nanostructures. For example, lipid-polymer hybrid nanoparticles combine the biocompatibility of lipids with the stability and modifiability of polymers, allowing for better control of drug release and increased therapeutic efficacy. Such systems can integrate multiple therapy functions or diagnostic and therapeutic effects (so-called theranostics), which is consistent with the development of medical nanobiotechnology as a tool for precision medicine [12,13].

As summarized in Table 1, the analytical and therapeutic performance of nanomaterials is strongly application-dependent. Inorganic nanomaterials generally offer superior analytical sensitivity and signal amplification, making them particularly suitable for diagnostics and imaging, whereas lipid-based systems demonstrate the highest biocompatibility and clinical readiness for drug and gene delivery. Polymeric and hybrid nanomaterials provide greater design flexibility and controlled release capabilities, positioning them as promising platforms for advanced theranostic and precision medicine applications.

From a translational perspective, different classes of nanomaterials exhibit distinct advantages and limitations that influence their clinical maturity. Lipid-based nanoparticles represent the most clinically advanced platform, as evidenced by their successful application in approved drug formulations and vaccines, due to their biocompatibility and scalable manufacturing [14]. Polymeric nanoparticles and dendrimers are characterized by high structural flexibility and tunable drug release profiles. Unfortunately, their clinical application is often limited by yield or control of synthesis [15]. Metallic and inorganic nanoparticles provide unique optical and magnetic properties that are particularly valuable in diagnostics and imaging, yet concerns related to long-term accumulation and potential toxicity have restricted their widespread clinical use [16]. Hybrid nanostructures aim to combine the favorable properties of different materials, but their increased structural complexity may pose additional regulatory and manufacturing challenges. These differences highlight that clinical applicability depends not only on therapeutic performance but also on safety, reproducibility, and regulatory feasibility.

### 2.2. Properties Determining Medical Applications

#### 2.2.1. Size, Shape, and Specific Surface Area

The size, shape, and surface-to-volume ratio of nanostructures are among the key parameters that determine their behavior in biological organisms and influence their medical applications. Nanomaterials typically have diameters ranging from a few to several hundred nanometers, allowing them to easily penetrate biological barriers and influencing their distribution in tissues. Smaller nanoparticles can be internalized by cells more quickly, while larger ones may have a longer circulation in the blood. The shape of nanoparticles (spherical, fibrous, platelet) also influences their interactions with cells and can modify bioavailability and immune clearance mechanisms. Furthermore, a large specific surface area increases the number of contact points with biological molecules and drugs, improving the efficiency of adsorption and therapeutic delivery [17]. It should be noted that the physicochemical parameters of nanoparticles described above may differ substantially from their effective properties in vivo due to the formation of a protein corona upon contact with biological fluids. Immediately after entering the bloodstream, nanoparticles adsorb plasma proteins, resulting in the formation of a dynamic protein layer that alters their apparent size, surface charge, and biological identity. This process can significantly influence biodistribution, cellular uptake, immune recognition, and targeting efficiency, often overriding the originally designed surface characteristics. Consequently, protein corona formation represents a critical factor in nanomedicine development and partly explains discrepancies between in vitro performance and in vivo outcomes. Surface functionalization strategies, such as PEGylation or biomimetic coatings, are commonly employed to modulate protein corona composition; however, complete control over this phenomenon under physiological conditions remains challenging [18,19].

#### 2.2.2. Biological Functionalization

Functionalization of nanomaterials refers to the modification of their surface by attaching various chemical groups, ligands, polymers, or biomolecules to impart the desired biological properties. Such surface modifications can improve solubility, target cell or receptor attachment, and modulate the immune response. For example, nanoparticles can be coated with polyethylene glycol (PEG) to prolong their circulation time in the blood and reduce immune recognition, while ligands such as peptides or antibodies can target nanostructures to specific cell types. Functionalization can also influence interactions with biomolecules and improve the stability of nanostructured systems under biological conditions, which is important for therapeutic or diagnostic applications [17].

#### 2.2.3. Biocompatibility and Stability

The biocompatibility of nanomaterials refers to their ability to interact with living systems without triggering toxic reactions. Good biocompatibility is crucial because nanostructures come into contact with blood, cells, and tissues, which can trigger an immune response or cytotoxicity if the materials are not properly selected. The stability of nanomaterials in the biological environment means that they do not undergo uncontrolled aggregation, degradation, or chemical alteration, which could impair their therapeutic or diagnostic function. Designing nanostructures with high biocompatibility and stability involves selecting materials that are well-tolerated by the body and surface strategies that minimize negative interactions with the immune system while ensuring proper drug release or signaling [20,21]. Numerous studies have demonstrated that nanoparticle physicochemical parameters critically determine therapeutic success or failure. For instance, nanoparticles with diameters below approximately 5–10 nm may undergo rapid renal clearance, resulting in insufficient accumulation at the target site, whereas larger particles may exhibit prolonged circulation but increased uptake by the mononuclear phagocyte system. Surface functionalization strategies such as PEGylation have been shown to extend blood circulation time and reduce immune recognition; however, excessive surface shielding can also impair cellular uptake and reduce intracellular drug delivery efficiency. In clinical and translational studies, these trade-offs have contributed to discrepancies between strong in vitro performance and limited in vivo or clinical efficacy. Furthermore, insufficient biocompatibility or the instability of nanocarriers under physiological conditions has, in some cases, led to unexpected toxicity or loss of therapeutic function, underscoring the importance of balanced nanoparticle design [22].

## 3. Drug Delivery and Targeted Therapy

### 3.1. Mechanisms of Targeted Drug Delivery

#### 3.1.1. Passive and Active Nanocarrier Targeting

**Figure 2:**
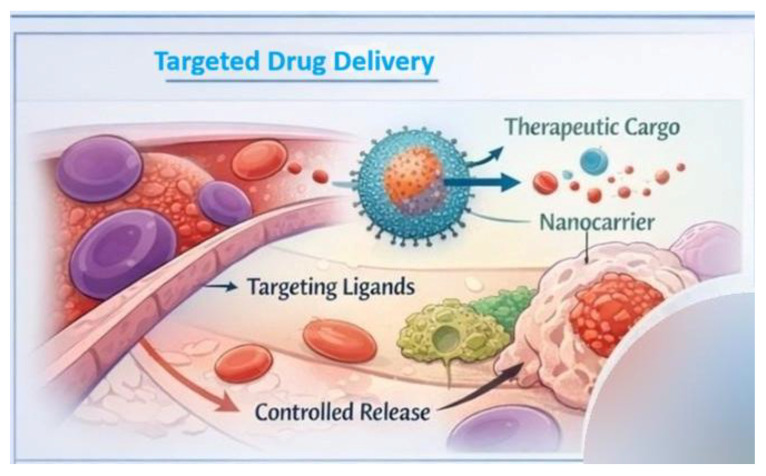
Applications of nanobiotechnology in medicine: nanocarrier-mediated targeted drug delivery and controlled release.

Targeted drug delivery using nanocarriers involves two main strategies: passive and active targeting. In passive targeting, nanoparticles accumulate at the disease site without any special modifications, most often due to the enhanced permeability and retention (EPR) effect. This effect occurs in pathological tissues, such as tumors, which have leaky blood vessels and inefficient lymphatic drainage, favoring the penetration and retention of nanocarriers of appropriate size. Passive targeting improves the biodistribution of the therapeutic and allows for higher concentrations in the target tissue while simultaneously reducing its presence in healthy cells [23,24]. Active targeting, on the other hand, utilizes targeted modifications to nanocarriers, such as the attachment of ligands that recognize specific receptors or structures on the surface of target cells (Figure 2). These ligands may be antibodies, peptides, folic acid, or other molecules that specifically bind to receptors overexpressed on cancer cells or in the disease area. This specialized recognition allows nanocarriers to be retained and internalized within target cells via receptor-mediated mechanisms, increasing therapeutic efficacy and reducing side effects [25,26]. The EPR effect has long been considered a central mechanism enabling the passive accumulation of nanocarriers in tumor tissue. While robust EPR-driven accumulation has been consistently demonstrated in murine tumor models, growing clinical evidence indicates that the magnitude and reliability of this effect in human cancers are highly variable and often limited. Tumor heterogeneity, differences in vascular permeability, interstitial fluid pressure, and lymphatic drainage significantly influence nanoparticle extravasation and retention in patients [27]. Clinical studies have shown that only a small fraction of systemically administered nanoparticles typically reaches the tumor site, and EPR-mediated delivery alone is frequently insufficient to achieve meaningful therapeutic benefit. As a result, overreliance on the EPR effect has been recognized as one of the factors contributing to the gap between promising preclinical nanomedicine results and modest clinical outcomes [28]. Consequently, current research efforts increasingly focus on strategies to improve or complement EPR-based delivery, including patient stratification, vascular modulation, active targeting approaches and local or image-guided administration. These developments reflect a shift from viewing the EPR effect as a universal principle toward considering it as a context-dependent phenomenon that must be carefully evaluated in clinically relevant settings [29].

#### 3.1.2. Controlled Release of Active Substances

The effectiveness of nanocarriers in drug delivery depends not only on their ability to reach the site of action but also on the ability to precisely release the active substance at the appropriate time and location. Nanocarriers can be designed to release therapeutics in a controlled manner, meaning that drug release occurs gradually or in response to specific biological or external signals. Modern controlled release (Figure 2) systems utilize so-called stimuli-responsive nanocarriers. These systems can release the drug in response to environmental changes characteristic of the disease microenvironment, such as reduced pH, high enzyme concentrations, reactive oxygen species, or redox gradients, or in response to external signals, such as temperature, light, magnetic fields, or ultrasound. This type of controlled drug distribution allows for drug concentration at the target site and minimizes uncontrolled release in healthy tissues [30,31]. In summary, the properties of nanocarriers, their targeted transport and controlled release are critical for achieving high therapeutic efficacy while limiting side effects, which is a promising perspective for the development of therapies based on nanobiotechnology [32]. Despite the conceptual advantages of controlled and stimuli-responsive drug release systems, their performance under in vivo conditions often differs from that observed in simplified in vitro models. Physiological variability in pH, enzyme activity, redox potential, and tissue perfusion can lead to unpredictable or premature drug release, reducing therapeutic precision [33]. Moreover, external stimulus-based systems, such as light-, heat-, or magnetically triggered nanocarriers, may face limitations related to tissue penetration depth, spatial control, and patient-to-patient variability [34]. From a translational perspective, additional challenges include the complexity of nanocarrier design, difficulties in large-scale manufacturing, and batch-to-batch reproducibility as well as regulatory hurdles associated with multifunctional and responsive systems. In several cases, controlled-release nanocarriers that demonstrated superior efficacy in preclinical models failed to show significant clinical advantages over conventional formulations, underscoring the need for the realistic evaluation of release mechanisms in clinically relevant environments [35,36].

### 3.2. Therapeutic Applications

#### 3.2.1. Oncology

**Figure 3:**
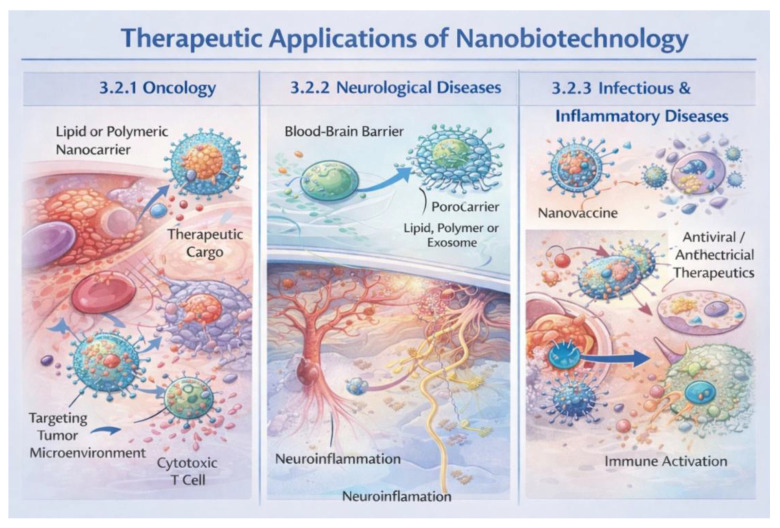
Schematic overview of the major therapeutic applications of nanobiotechnology, illustrating nanocarrier-based strategies in oncology, neurological disorders, and infectious and inflammatory diseases. The figure highlights targeted drug delivery to the tumor microenvironment, blood–brain barrier penetration for neurological applications, and nanotechnology-enabled immunomodulatory and anti-infective therapies.

Nanobiotechnology has found particularly widespread application in oncology, where nanosystems are used to improve the efficacy and safety of anticancer therapies. Nanocarriers enable the selective delivery of cytotoxic drugs to tumor tissue, thereby limiting their distribution to healthy organs. Due to the enhanced permeability of tumor vasculature and the possibility of active targeting, nanoparticles can accumulate within tumors and release therapeutic agents in a controlled manner (Figure 3). This approach allows for higher drug concentrations at the disease site, reduced systemic toxicity, and improved treatment tolerability. In addition, nanomaterials are being investigated as components of combination therapies, integrating chemotherapy with photothermal therapy or immunotherapy [37].

#### 3.2.2. Neurological Diseases

The use of nanobiotechnology in the treatment of neurological diseases stems primarily from the need to effectively overcome the blood–brain barrier (Figure 3), which poses a significant limitation for conventional drugs. Properly designed nanoparticles can transport therapeutic substances such as neuroprotective drugs, peptides, or nucleic acids into the central nervous system. Functionalizing the nanocarrier surface with ligands that recognize transporters or receptors present on endothelial cells enables their active transport across the blood–brain barrier. These strategies are being explored in the context of treating neurodegenerative diseases such as Alzheimer’s and Parkinson’s disease as well as in the therapy of brain tumors [38,39]. Despite the extensive body of preclinical data demonstrating the high efficacy of nanocarrier-based drug delivery systems in oncology and neurological disorders, the translation of these results into consistent clinical success remains limited. In preclinical cancer models, actively targeted nanocarriers functionalized with antibodies, peptides, or small-molecule ligands often show enhanced tumor accumulation, increased cellular uptake, and superior antitumor efficacy compared to non-targeted formulations. However, clinical studies frequently reveal only marginal improvements over conventional therapies or passively targeted nanocarriers. This discrepancy can be largely attributed to the oversimplified nature of preclinical models, which fail to fully reproduce tumor heterogeneity, stromal barriers, abnormal interstitial pressure, and the dynamic vascular architecture observed in human tumors [40]. Additionally, the formation of a protein corona under physiological conditions can significantly alter the surface properties of actively targeted nanocarriers, masking targeting ligands and reducing receptor-specific interactions in vivo. Interpatient variability in receptor expression, immune clearance, and pharmacokinetics further limits the predictability of active targeting strategies at the clinical level. Consequently, while active targeting enhances mechanistic understanding and cellular-level interactions, its impact on clinical outcomes has so far been less pronounced than anticipated based on preclinical evidence. Similar translational challenges are observed in neurological applications, particularly for nanocarriers designed to penetrate the blood–brain barrier (BBB). Numerous preclinical studies report efficient BBB crossing through receptor-mediated transcytosis or adsorptive transport mechanisms, leading to improved drug accumulation in the brain and neuroprotective or antitumor effects. In contrast, clinical translation is hindered by substantial interspecies differences in BBB structure, transporter expression, and endothelial cell biology, which limit the relevance of commonly used animal models. Moreover, achieving therapeutically meaningful drug concentrations in the human brain often requires dosing levels that raise concerns regarding systemic toxicity and long-term safety [41]. Taken together, these observations highlight a persistent gap between preclinical efficacy and clinical outcomes in both oncological and neurological nanomedicine. Future progress will depend on the development of more predictive preclinical models, improved characterization of nano–bio interactions in human systems, and a stronger focus on clinically relevant endpoints rather than solely on enhanced accumulation or cellular uptake. Addressing these challenges is essential for the successful translation of advanced nanocarrier systems from experimental research to routine clinical practice.

#### 3.2.3. Infectious and Inflammatory Diseases

Nanobiotechnology is playing an increasingly important role in the treatment of infectious and inflammatory diseases, offering new strategies for delivering antibacterial, antiviral, and anti-inflammatory drugs (Figure 3). Nanocarriers can increase the stability and bioavailability of antibiotics and enable their targeted delivery to sites of infection, which is particularly important in the context of increasing microbial drug resistance. In inflammatory diseases, nanosystems can modulate the immune response by selectively delivering drugs to immune cells or inflamed tissues. Nanoparticles are also being explored as platforms for modern vaccines and antiviral therapies, which has gained particular importance in recent years [42,43].

## 4. Nanobiotechnology in Diagnostics and Medical Imaging

### 4.1. Molecular Nanodiagnostics

Molecular nanodiagnostics utilizes nanomaterials and structures that can precisely detect and measure specific biological molecules indicative of a disease state. In this context, nanotechnology-based biosensors, also known as nanobiosensors, integrate biological recognition elements (such as antibodies, aptamers, or enzymes) with nanostructures that convert the biological signal into a detectable electrical, optical, or magnetic signal. Nanomaterials such as gold nanoparticles, carbon nanotubes, or quantum dots enable increased detection sensitivity and lowered analyte detection thresholds, even to very low concentrations, which is crucial for diagnosing diseases at an early stage [44,45]. Nanotechnology-based biosensors can function as miniature lab-on-a-chip systems capable of rapidly analyzing biological samples with minimal material, making them promising tools in both scientific research and clinical diagnostics. Integrating nanostructures with biological components allows for the generation of signals with high specificity and selectivity, enabling the identification of molecules even in complex biological samples such as blood or urine [46]. Detection of biomarkers—biological molecules whose presence or altered concentration indicates pathology—is one of the main goals of molecular nanodiagnostics. Nanostructures such as quantum dots or magnetic nanoparticles can be conjugated with biological receptors, allowing them to recognize and bind to target biomarkers such as cancer proteins, nucleic acids, or pathogen fragments. Upon target binding, the nanostructure generates an optical, magnetic, or electrical signal proportional to the amount of the detected biomarker, enabling highly sensitive and rapid detection [47,48]. This approach is particularly valuable in early disease detection, before clinical symptoms develop, which increases the chances of effective treatment. For example, nanodiagnostics allows for the identification of tumor markers in the blood at very low levels, which can lead to earlier cancer diagnosis and improved treatment outcomes [49]. Nanodiagnostic and biosensor-based technologies encompass a broad spectrum of development stages, ranging from clinically approved systems to platforms that remain primarily within the research and preclinical validation phase. Several nanotechnology-enabled diagnostic approaches have already reached clinical implementation, particularly in the form of nanoparticle-based contrast agents used in medical imaging. Iron oxide nanoparticles for magnetic resonance imaging and gold-based nanomaterials employed in computed tomography or optical imaging represent established examples of nanodiagnostics integrated into routine clinical workflows. In addition, lipid nanoparticle-based platforms enabling nucleic acid detection and delivery have gained regulatory approval in specific diagnostic and therapeutic contexts, underscoring the clinical feasibility of nanoscale diagnostic systems [50]. In contrast, the majority of nanobiosensors and molecular nanodiagnostic platforms remain at the experimental or early translational stage. Highly sensitive nanosensors based on quantum dots, carbon nanotubes, graphene, or plasmonic nanoparticles demonstrate exceptional analytical performance under laboratory conditions; however, their clinical translation is often limited by challenges related to long-term stability, reproducibility, scalability of fabrication, and regulatory validation. Furthermore, the integration of such nanosensors into standardized diagnostic workflows requires robust validation in large patient cohorts and compliance with strict regulatory requirements, which remains a significant barrier to widespread clinical adoption [51]. In the context of infectious diseases, nanodiagnostic platforms have shown particular promise for rapid and point-of-care detection of pathogens and biomarkers. While several nanoparticle-assisted assays and lab-on-a-chip systems have been evaluated in clinical pilot studies, only a limited number have progressed to routine clinical use. Most platforms are still undergoing optimization to balance sensitivity, specificity, cost-effectiveness, and operational simplicity. Therefore, despite substantial advances at the research level, the translational pathway of nanodiagnostics continues to be constrained by technical, regulatory, and economic factors.

Overall, the current landscape of nanodiagnostics reflects a clear distinction between clinically implemented nanomaterial-based imaging agents and emerging biosensor technologies that, although highly promising, largely remain within the research and early translational domains. Bridging this gap will require standardized validation protocols, scalable manufacturing strategies, and closer alignment between technological development and clinical needs.

### 4.2. Application of Nanomaterials in Imaging

Fluorescence bioimaging utilizes light-emitting nanomaterials that, when excited at a specific wavelength, can be detected by optical detectors, enabling the visualization of biological structures with high sensitivity and resolution. Fluorescent nanoparticles, such as quantum dots or lanthanoid-based nanoparticles, can be functionalized to specifically bind to specific biomolecules or cells, allowing for precise localization within a biological system. Due to their unique optical properties, such as high brightness, resistance to photoblocking, and the ability to tune the wavelength of light emission, fluorescent nanoparticles outperform conventional organic dyes in molecular bioimaging applications. They are particularly useful for imaging dynamic biological processes and detecting disease-related molecular changes [52]. Fluorescence bioimaging using nanomaterials also enables the analysis of intracellular processes and real-time monitoring of therapy. Nanoparticles can be designed to emit light in the near-infrared (NIR) range, which reduces tissue autofluorescence and allows for deeper light penetration into the body, which is useful in the analysis of living organisms and tissues [53].

**Figure 4:**
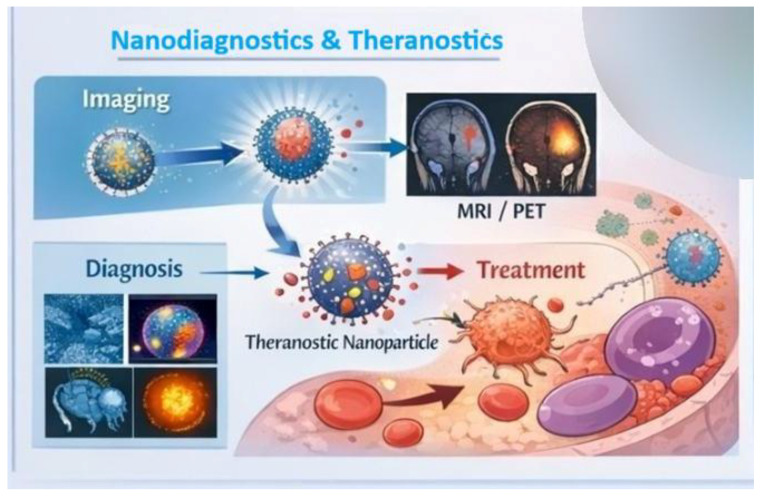
Applications of nanobiotechnology in medicine: nanodiagnostic and theranostic platforms for imaging and treatment.

Nanomaterials are widely used as modern contrast agents in various medical imaging techniques (Figure 4), improving the visibility of anatomical structures and physiological processes. In magnetic resonance imaging (MRI), magnetic nanoparticles such as iron oxide nanoparticles can enhance signal contrast by altering proton relaxation in tissues, facilitating the detection of pathological changes [54]. Nanomaterials can also act as contrast agents in X-ray (CT) imaging and other modalities due to their unique physical properties. For example, metallic nanoparticles with high electron density, such as gold nanoparticles, absorb X-ray radiation more strongly than traditional contrast agents, allowing for clearer visualization of anatomical structures and lesions [55]. Furthermore, nanomaterials can be designed to function in several imaging techniques simultaneously (so-called multimodal imaging), enabling the acquisition of more comprehensive diagnostic information from a single imaging agent. For example, nanomaterials can integrate MRI signals with optical or PET signals (Figure 4), allowing the study of pathological changes at different scales and with different types of information [56].

### 4.3. Nanobiotechnology-Based Sensing and Biosensor Applications

**Figure 5:**
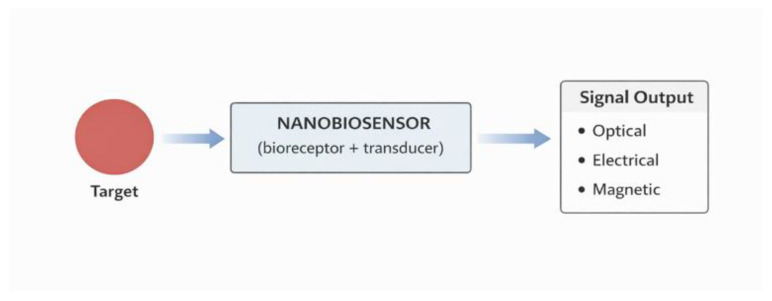
Schematic representation of a nanobiosensor system, illustrating the interaction between the target analyte and the nanobiosensor composed of a bioreceptor and a transducer, followed by signal conversion into optical, electrical, or magnetic outputs.

Nanobiotechnology-based sensing represents one of the most rapidly emerging domains within modern diagnostics, driven by the demand for rapid, sensitive, and point-of-care detection of clinically relevant biomarkers. Nanobiosensors (Figure 5) integrate biological recognition elements, such as antibodies, aptamers, enzymes, or nucleic acids, with nanostructured transducers that convert biological interactions into measurable optical, electrical, or electrochemical signals. The nanoscale dimensions of these systems enable enhanced surface-to-volume ratios, improved signal amplification, and ultra-sensitive detection capabilities compared to conventional sensing platforms [57]. Among the most established nanobiosensing platforms are gold nanoparticle-based sensors, which exploit localized surface plasmon resonance (LSPR) for optical detection. These systems are widely used in lateral flow assays and colorimetric biosensors, where target binding induces measurable changes in optical properties. Such platforms have been successfully applied in infectious disease diagnostics, including viral and bacterial detection, due to their simplicity, rapid response, and compatibility with point-of-care testing [58]. Carbon-based nanomaterials, including graphene and carbon nanotubes, have also gained considerable attention in biosensing applications due to their exceptional electrical conductivity and sensitivity to surface charge changes [59]. Graphene-based field-effect transistor (FET) biosensors enable the label-free, real-time detection of biomolecules at extremely low concentrations and are being explored for the detection of pathogens, nucleic acids, and protein biomarkers [60].

Despite their high analytical performance, these systems remain largely at the research stage due to challenges related to device fabrication, reproducibility, and large-scale integration. Magnetic nanoparticle-based sensors constitute another important class of nanobiosensors, particularly in molecular diagnostics. Magnetic nanoparticles can be functionalized with biological probes to selectively capture target analytes, enabling signal amplification and separation from complex biological matrices. These systems are commonly integrated with nucleic acid amplification techniques and are increasingly used in automated diagnostic platforms [61]. In addition, emerging sensing approaches involve quantum dots and other fluorescent nanomaterials, which provide high photostability, tunable emission spectra, and multiplexing capabilities [62]. These properties make them attractive for the simultaneous detection of multiple biomarkers in complex samples. However, concerns related to toxicity and acceptance continue to limit their clinical translation. Nanobiotechnology-based sensing offers transformative potential for next-generation diagnostics by enabling rapid, sensitive, and decentralized testing. Nevertheless, further efforts are required to address challenges related to standardization, scalability, and regulatory validation to facilitate broader clinical implementation.

## 5. Tissue Engineering and Regenerative Medicine

### 5.1. Nanostructured Biomedical Scaffolds

**Figure 6:**
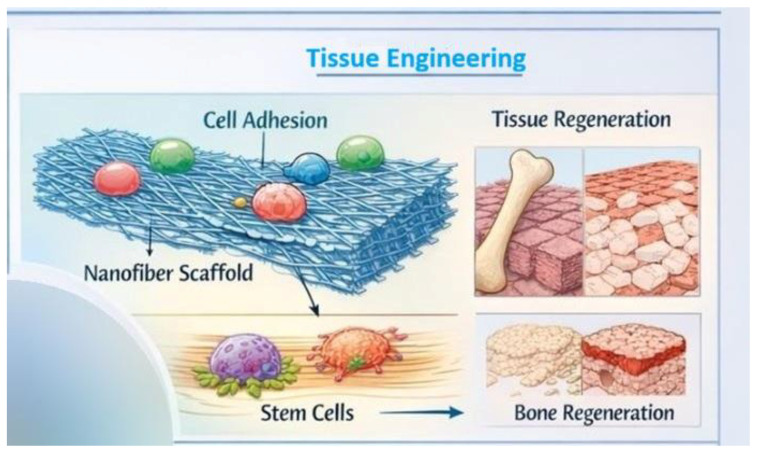
Applications of nanobiotechnology in medicine: nanofiber scaffolds for tissue engineering and regeneration.

In tissue engineering, it is crucial to develop structures that mimic the nature of the extracellular matrix (ECM)—a network of proteins and polysaccharides that creates a physical and biological environment for cells. Nanostructured biomedical scaffolds are designed to have a fibrous architecture comparable in size to collagen fibers in the ECM, providing a similar mechanical and spatial environment for tissue growth (Figure 6). Such structures promote cell migration, integration, and new tissue formation because their nanoscale components mirror the natural characteristics of the ECM, facilitating cell recognition and adaptation to the environment [63,64]. Nanostructured scaffolds can be fabricated using methods such as electrospinning, self-assembly, or phase separation to obtain porous, three-dimensional fiber structures with diameters similar to those of natural ECM components. This allows cells to feel more like they are in their physiological microenvironment, promoting their adhesion, proliferation, and organization into tissue-like structures [64]. Nanostructured scaffolds not only physically mimic the ECM but also actively influence cell behavior. Their high surface area and appropriate topography facilitate cell adhesion, which is essential for the stable development of new tissue. Through mechanical and biological interactions between cells and nanofibers, signals transmitted to the cell cytoskeleton can modulate cell behavior, leading to improved proliferation and differentiation into target cells. Nanostructures can also deliver growth factors or bioactive molecules, which further direct the differentiation of stem cells into specific cell lineages essential for tissue regeneration, such as osteoblasts, fibroblasts, or neurons [65,66]. Studies have shown that various nanostructure parameters, such as fiber orientation, diameter, and pore distribution, influence the direction of cell migration and their morphology. For example, fibers with a specific arrangement can promote directed cell migration and support their differentiation into preferred lineages, which is particularly important in the regeneration of complex tissues such as nerves or muscles [67]. Despite the significant progress in the development of nanostructured scaffolds for tissue engineering (Figure 6) and regenerative medicine, their widespread clinical implementation remains limited by several critical challenges. One of the primary concerns is the long-term stability of nanostructured scaffolds under physiological conditions. Degradation kinetics, mechanical integrity, and the preservation of nanoscale architecture over extended periods are crucial parameters that influence both tissue integration and functional outcomes. In vivo degradation products must also be carefully evaluated to ensure biocompatibility and to avoid adverse inflammatory or immunological responses [68]. From a clinical perspective, regulatory approval represents a substantial barrier to translation. Nanostructured scaffolds often combine characteristics of medical devices, biomaterials, and, in some cases, drug delivery systems, which complicates their regulatory classification and evaluation. The lack of standardized guidelines specific to nanoscale scaffolds further hampers regulatory assessment, as traditional testing frameworks may not adequately capture nano-specific properties such as surface topology, nanoscale roughness, or bioactive functionalization. Manufacturing and scalability pose additional challenges for clinical translation. Techniques commonly used to fabricate nanostructured scaffolds, including electrospinning, self-assembly, and advanced 3D printing, can be difficult to reproduce with high batch-to-batch consistency when scaled up for industrial production. Maintaining precise control over fiber diameter, pore size distribution, mechanical properties, and bioactive molecule incorporation at large scale remains technically demanding. Variability in scaffold architecture can directly impact cellular responses and therapeutic outcomes, underscoring the importance of reproducibility and quality control [69]. Overall, successful clinical translation of nanostructured scaffolds will require the integration of robust manufacturing protocols, standardized characterization methods, and regulatory frameworks specifically adapted to nanostructured biomaterials. Addressing these challenges is essential to bridge the gap between laboratory-scale innovation and reliable, large-scale clinical application in regenerative medicine.

### 5.2. Clinical Applications

**Figure 7:**
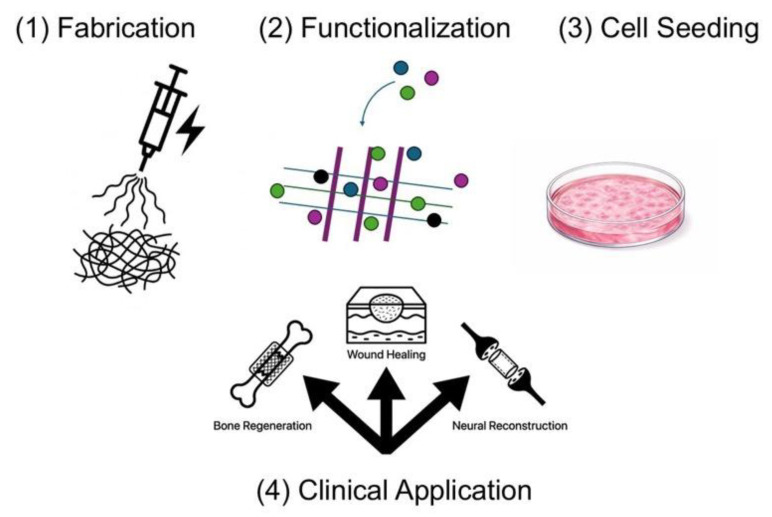
Schematic of the fabrication process and clinical application of nanostructured scaffolds. The process includes: (**1**) fabrication of a fibrous mat (e.g., via electrospinning) mimicking the extracellular matrix (ECM), (**2**) functionalization with growth factors, (**3**) in vitro cell seeding and proliferation, and (**4**) therapeutic application in bone regeneration, wound healing (skin), and nerve reconstruction.

In the context of bone regeneration, nanobiotechnology opens new therapeutic possibilities that surpass traditional approaches to treating bone defects, such as autografts or allografts. Nanomaterials can be used to fabricate biocompatible scaffolds and nanosystems that promote the proliferation and differentiation of osteogenic cells, as well as angiogenesis—the formation of new blood vessels—which is crucial for the integration of newly formed tissue into the host. Hydroxyapatite nanoparticles or composite nanostructures combined with polymers can improve the mechanical and biological properties of implants, resulting in more efficient bone regeneration and enhanced integration with surrounding tissue [70]. Skin healing and soft tissue regeneration also benefit significantly from nanotechnology-based approaches. Nanomaterials can function as drug carriers, facilitating the delivery of bioactive molecules to the wound site, accelerating the healing process (Figure 7), and reducing the risk of infection. In clinical practice, nanostructures in the form of hydrogels or nanofibrillar scaffolds are used in the treatment of chronic wounds, burns, and ulcers. Their microporous architecture mimics the extracellular matrix and supports skin cell migration and new tissue formation [71]. Furthermore, nanobiotechnology contributes to enhanced angiogenesis and modulation of the immune response, which is particularly important for wounds that heal slowly or in patients with metabolic disorders. Certain nanomaterials also exhibit antibacterial properties, further reducing the risk of infection during soft tissue regeneration [72]. Nanobiotechnology in reconstructive medicine encompasses a broad spectrum of applications aimed not only at rebuilding tissue-deficient structures but also at restoring their function and anatomy, often following trauma, surgery, or in the case of congenital defects. Nanostructures are designed to integrate with the body’s natural biological processes by providing coordinated molecular and mechanical cues. For example, in cervical, cranial, and facial bone reconstruction, osteoconductive and osteoinductive nanomaterials can support bone growth and repair defects (Figure 7) [73]. In implantology and joint reconstruction, nanolayered implant coatings have been shown to enhance tissue adhesion, reduce the risk of implant rejection, and improve long-term durability. This approach may significantly decrease the need for reoperations and improve patient quality of life. Nanomaterials are also applied in soft and neural tissue reconstruction (Figure 7), where biomimetic structures and therapeutic carriers support cell growth and reduce scar formation. In addition, nanobiotechnology-based tools are being developed for cell-based therapies and combined growth factor delivery systems, enabling precise spatial and temporal control over the regeneration process at the site of injury—an aspect that is particularly important in the treatment of complex anatomical defects [72].

## 6. Advanced Therapies: Gene Therapy and Immunotherapy

**Figure 8:**
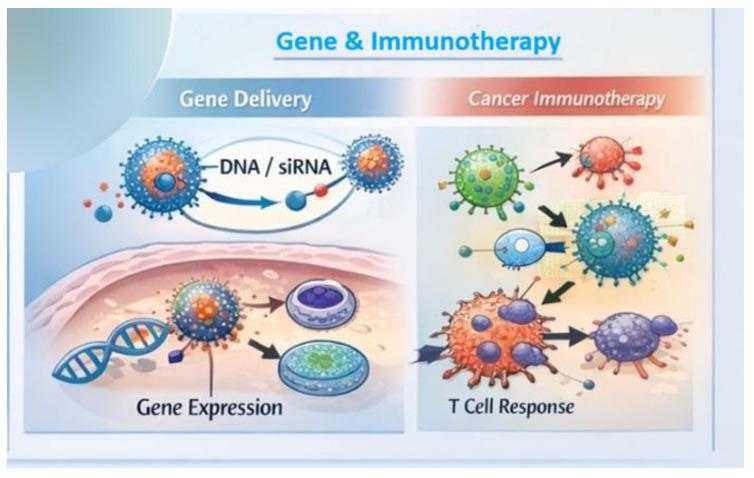
Applications of nanobiotechnology in medicine: nanotechnology-enabled gene delivery and cancer immunotherapy.

Nanocarriers designed for gene therapy primarily serve the safe and efficient transport of genetic material into target cells. This material can include various forms of nucleic acids: plasmid DNA (pDNA), mRNA, small interfering RNA (siRNA), or antisense oligonucleotides, which are intended to modulate gene expression or introduce new biological functions (Figure 8). Nanostructures such as lipid, polymer, or biomimetic nanoparticles (e.g., exosomes) are designed to effectively protect the genetic payload and enable its internal transport to the appropriate location within the cell, e.g., the cytoplasm or nucleus. Surface modifications of nanocarriers also enable targeted delivery to specific cell types within the body, increasing therapy efficacy and reducing side effects [74,75]. Nanocarrier research encompasses various delivery strategies, from non-viral carriers (lipid, polymeric) to biomimetic systems, which aim to improve the intracellular availability and translation of genetic materials and enable RNA-based therapy, e.g., through RNA interference or therapeutic mRNA [76].

One of the main challenges of gene therapy is that free nucleic acids are rapidly degraded by nucleases in the circulation and within cells, significantly limiting their therapeutic efficacy. Nanocarriers act as protective shields, encapsulating DNA, RNA, or oligonucleotides within their structure, thus protecting them from enzymatic degradation and improving their stability in the biological environment. Nanocarriers can also facilitate transport across cell membranes and escape from endosomes, which is crucial because genetic material must reach the site of action before being degraded [75,77]. Furthermore, designing the surface of nanocarriers, e.g., through sources of chemical modifications or loading lipids and polymers in an appropriately balanced manner, optimizes the protection of genetic material and facilitates its release in intracellular conditions in a controlled manner, which increases the effectiveness of gene therapy [78].

### 6.1. Nanobiotechnology in Modulating the Immune Response

Nanotechnology is transforming the approach to vaccine design, offering tools that improve their stability, accurate antigen delivery, and immunostimulatory potential. Nanovaccines are vaccines that utilize nanocarriers that encapsulate antigens and, often simultaneously, adjuvants, contributing to a stronger and more specific immune response. Nanocarriers can protect antigens from premature degradation and target them to professional antigen-presenting cells, such as dendritic cells, promoting more effective stimulation of both humoral and cellular immune responses. These platforms are being explored not only as preventive vaccines against infections but also as therapeutic anticancer vaccines, aiming to enhance the immune system’s ability to recognize and eliminate cancer cells [79,80]. Nanovaccines represent a promising platform because nanosystem properties such as size, surface area, and modifiability can be tailored to optimize antigen presentation, immune cell activation, and cargo release. Many researchers emphasize that nanotechnology can enable a broader spectrum of immune responses with a smaller antigen dose, potentially improving vaccine efficacy and reducing adverse events [81].

Nanobiotechnology is playing an increasingly important role in immunotherapy, a therapy that modulates the immune system to combat diseases such as cancer, infections, or autoimmune diseases. Nanostructures can be designed to precisely deliver immunomodulators such as cytokines, receptor ligands, checkpoint blockers, or peptides directly to specific immune cells or the tumor microenvironment, minimizing systemic toxicity [82]. Examples include nanoparticles functionalized to influence antigen presentation, activate T cells, or modify the tumor microenvironment, which otherwise inhibits the immune response. Nanomaterials can also support the penetration of cytotoxic T cells into inaccessible tumor areas and improve their ability to eliminate cancer cells (Figure 8) [83]. Furthermore, immunomodulatory nanostructured systems are being investigated for applications not only in oncology but also in infectious diseases, autoimmune diseases, and inflammation, where modulating the immune response can lead to improved therapeutic outcomes. Nanomaterials can both enhance targeted immune stimulation (e.g., in cancer therapy) and suppress excessive immune responses (e.g., in autoimmune diseases), making them versatile therapeutic tools in medicine [84].

### 6.2. Delivery Barriers and Design Strategies in Gene and Immunotherapy Nanocarriers

The clinical translation of gene therapy and immunotherapy nanocarriers is strongly influenced by multiple biological delivery barriers that limit therapeutic efficacy (Figure 8). One of the most critical challenges is endosomal entrapment following cellular uptake. After internalization via endocytosis, a substantial fraction of nanocarriers becomes sequestered within endo-lysosomal compartments, where acidic conditions and enzymatic activity can degrade nucleic acids or protein-based therapeutics before they reach their intracellular targets. To address this limitation, current nanocarrier designs frequently incorporate pH-responsive or membrane-disruptive components that facilitate endosomal escape, such as ionizable lipids, proton-sponge polymers, or fusogenic peptides that destabilize endosomal membranes under acidic conditions [85]. Immune recognition represents another major barrier, particularly for repeated administration of nanocarrier-based therapeutics. Nanoparticles can be rapidly opsonized by serum proteins, leading to accelerated clearance by the mononuclear phagocyte system and reduced bioavailability. Moreover, innate immune activation may result in inflammatory responses or neutralization of the therapeutic payload. To mitigate these effects, surface engineering strategies such as polyethylene glycol (PEG)ylation, zwitterionic coatings, or biomimetic camouflage using cell membrane-derived materials are employed to reduce immune recognition and prolong circulation time. These approaches aim to balance immune evasion with sufficient cellular uptake at the target site [86].

Off-target effects further complicate the therapeutic use of gene and immunotherapy nanocarriers, as unintended biodistribution can lead to gene expression or immune modulation in non-target tissues. Such effects raise safety concerns and limit the therapeutic window. To improve targeting specificity, nanocarriers are increasingly functionalized with ligands that recognize cell-type-specific receptors or are engineered to respond to disease-associated stimuli, such as pH, enzymatic activity, or redox gradients. These strategies promote localized cargo release and reduce systemic exposure [87]. Overall, advances in nanocarrier design increasingly focus on integrating multiple functional features—such as immune stealth, controlled intracellular trafficking, and stimulus-responsive release—within a single platform. While these approaches have significantly improved delivery efficiency at the preclinical level, their clinical translation remains dependent on achieving an optimal balance between efficacy, safety, and manufacturability. A deeper understanding of nano–bio interactions in human systems will be essential for further progress in gene therapy and immunotherapy applications.

## 7. Safety, Toxicity, and Regulatory Challenges

**Figure 9:**
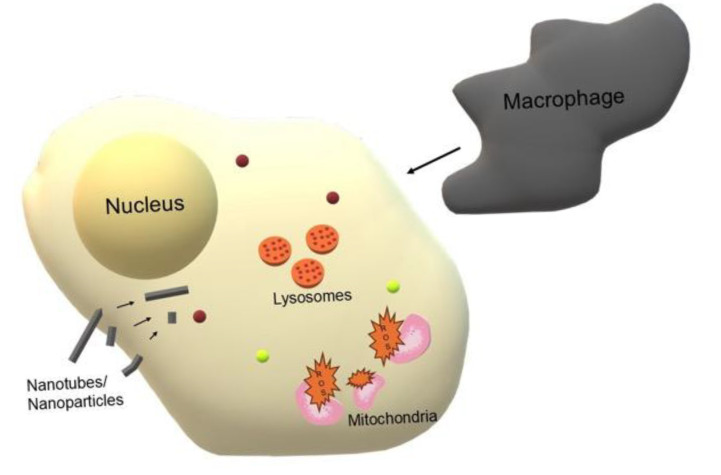
Potential pathways of cytotoxicity and immune response to nanomaterials. The schematic illustrates how the unique properties of nanomaterials enable cell penetration, which can lead to cellular dysfunction via oxidative stress (ROS generation in damaged mitochondria), organelle damage, and the induction of an inflammatory response.

Nanomaterials used in medicine have the potential to offer innovative therapeutic solutions, but their unique physicochemical properties can also lead to unintended toxic effects. Their reduced size and large surface area allow these particles to easily penetrate cells and tissues, where they can cause oxidative stress (by generating reactive oxygen species, ROS), inflammation, or damage to cellular organelles (Figure 9). Furthermore, various types of nanomaterials, particularly metallic and metal-oxide nanomaterials, can accumulate in organs such as the liver, spleen, and brain, raising concerns about their long-term health effects and potential glial or neurodegenerative consequences. In many cases, standard toxicological tests for small molecules are insufficient, highlighting the need to develop new methods for assessing safety specific to nanostructures [88,89].

Nanomaterials can have complex interactions with the immune system, which can be both beneficial and harmful. Particles can be recognized as foreign, leading to immune cell activation, cytokine production, and inflammation, and in extreme cases, hypersensitivity or immunotoxicity. Manipulating the surface properties of nanomaterials can enable controlled modulation of the immune system, which is used in immunotherapy and vaccines. However, subtle changes in the size, charge, or surface coverage of particles can significantly alter their immunological profile, making the assessment of these interactions a key element of preclinical research [90,91].

Regulations regarding medical nanomaterials are currently fragmented and often fail to keep pace with the rapid development of nanobiotechnology. The current regulatory framework in many countries is based on regulations for traditional drugs and medical devices, which do not always adequately address the unique properties of nanostructures, such as their dynamic interactions with the biological microenvironment or different pharmacokinetic profiles. Nanomaterials can be classified as drugs or medical devices, leading to discrepancies in regulatory requirements across regions. Moreover, the lack of precise metrological standards and the lack of consensus on methods for assessing safety and effectiveness hinder the process of clinical authorization and implementation into medical practice [92,93].

One of the main limitations in the translation of nanomaterials from the laboratory to the clinic is the lack of uniform research standards and methods for physicochemical and biological characterization. Different studies use different measurement methods and experimental protocols, making it difficult to compare results and generate consistent data on safety and efficacy. Standardization is crucial for assessing key quality attributes of nanomaterials such as the size, shape, stability under biological conditions and immunological properties. Without uniform standards and widely accepted guidelines for toxicological testing and risk assessment methods, the translation of innovative nanosystems into clinical practice remains slow, and their full therapeutic potential remains underutilized [93].

### Toxicological Concerns and Regulatory Pathways in Clinical Nanomedicine

While nanobiotechnology offers substantial therapeutic and diagnostic advantages, numerous nanomaterials have been associated with well-documented toxicological concerns that continue to limit their clinical translation. Metallic and metal-oxide nanoparticles, such as silver, gold, and iron oxide nanoparticles, have been reported to induce oxidative stress, mitochondrial dysfunction, and inflammatory responses, particularly following long-term or high-dose exposure [94]. For example, silver nanoparticles exhibit antimicrobial efficacy but may accumulate in organs such as the liver and spleen, raising concerns regarding cytotoxicity and genotoxicity. Similarly, iron oxide nanoparticles used as magnetic resonance imaging contrast agents can disrupt iron homeostasis and promote reactive oxygen species generation under certain conditions [95]. Carbon-based nanomaterials, including carbon nanotubes and graphene derivatives, have also demonstrated potential toxicological risks related to their high aspect ratio, persistence in tissues, and pro-inflammatory effects, which in some cases resemble asbestos-like pathogenicity. These materials may induce granuloma formation, fibrosis, or chronic inflammation, particularly following inhalation or systemic exposure. In polymeric nanocarriers, toxicity is often linked not to the polymer backbone itself but to degradation products, residual solvents, or cationic surface charges, which can cause membrane disruption and complement activation [96]. From a regulatory perspective, nanomedicine products are currently evaluated within existing pharmaceutical and medical device frameworks established by regulatory agencies such as the U.S. Food and Drug Administration (FDA) and the European Medicines Agency (EMA). Several nanomedicine formulations have successfully obtained regulatory approval, providing practical reference points for translational development. Clinically approved examples include liposomal formulations such as pegylated liposomal doxorubicin and lipid nanoparticle-based systems for nucleic acid delivery, which have demonstrated improved safety profiles compared to conventional formulations. These approvals illustrate that nanoscale drug formulations can meet regulatory standards when supported by comprehensive physicochemical characterization, validated manufacturing processes, and robust safety data [97]. However, regulatory assessment of nanomedicines remains challenging due to the absence of harmonized, nano-specific guidelines addressing parameters such as particle size distribution, surface chemistry, aggregation behavior, and in vivo transformation. Both the FDA and EMA increasingly emphasize a case-by-case, risk-based approach, requiring detailed evaluation of nano–bio interactions, biodistribution, and long-term toxicity. As a result, early integration of regulatory considerations into nanomaterial design and preclinical testing is essential to facilitate clinical translation and reduce developmental risk. Several nanomedicine products have successfully navigated these regulatory pathways and reached clinical approval, providing a practical framework for translation. Examples include liposomal formulations such as Doxil^®^ (pegylated liposomal doxorubicin) and AmBisome^®^ (liposomal amphotericin B), as well as lipid nanoparticle-based mRNA vaccines, which have demonstrated that nanoscale drug delivery systems can meet the regulatory requirements for safety, efficacy, and large-scale manufacturing [1,3,75,76]. These approved products illustrate that regulatory acceptance of nanomedicines is feasible, provided that comprehensive preclinical and clinical data are available. Importantly, toxicological concerns and regulations further emphasize the need for balanced analytical performance characterization of nanomaterials in various biomedical applications.

## 8. Comparative Evaluation of Nanomaterials Performance Across Biomedical Applications

The wide range of biomedical applications of nanomaterials underscores the need to critically compare the analytical performance of different classes of nanomaterials to identify their advantages and limitations. Analytical performance is understood as a multidimensional concept encompassing sensitivity, selectivity, stability, reproducibility, biocompatibility, and translational feasibility. The importance of these parameters varies significantly depending on the specific application, such as diagnostics, imaging, drug delivery, or theranostics. In diagnostic and biosensing applications, inorganic nanomaterials generally demonstrate superior analytical performance. Metallic nanoparticles, particularly gold nanoparticles, exhibit strong signal amplification properties arising from localized surface plasmon resonance, which enables highly sensitive optical and colorimetric detection [98]. These properties have been successfully exploited in lateral flow assays, optical biosensors, and point-of-care diagnostic platforms. Similarly, magnetic nanoparticles offer high analytical performance through magnetic signal amplification, efficient analyte separation, and compatibility with automated diagnostic systems [99].

Their ability to operate in complex biological matrices with minimal background interference makes them particularly suitable for molecular diagnostics and bioanalytical assays.

Semiconducting nanoparticles, such as quantum dots, further extend the analytical capabilities in fluorescence-based imaging and sensing [100].

Their high photostability, narrow emission spectra, and tunable optical properties allow for multiplexed detection and long-term imaging with minimal signal degradation. From a purely analytical perspective, quantum dots often outperform organic fluorophores and polymer-based probes. However, despite their excellent sensitivity and signal stability, concerns related to heavy metal toxicity and long-term accumulation have limited their clinical translation. Consequently, their superior analytical performance does not necessarily translate into superior overall biomedical performance.

Carbon-based nanomaterials, including graphene, graphene oxide, and carbon nanotubes, represent another class with outstanding analytical potential, particularly in electrochemical and field-effect transistor (FET)-based biosensors [101].

Their large specific surface area, exceptional electrical conductivity, and sensitivity to surface charge changes enable ultra-low detection limits and real-time signal transduction. These features make carbon nanomaterials highly attractive for next-generation biosensing platforms [102]. Nevertheless, their analytical performance is often accompanied by challenges related to batch-to-batch reproducibility, surface heterogeneity, and variability in biological interactions, which can complicate standardization and regulatory approval. In contrast to inorganic and carbon-based nanomaterials, lipid-based and polymeric nanocarriers are engineered for therapeutic delivery and biocompatibility, and therefore do not possess intrinsic imaging or electrochemical signal properties [103]. However, their performance should be evaluated using fundamentally different criteria. In drug delivery, gene therapy, and immunotherapy applications, analytical performance is closely linked to delivery efficiency, biological response, and therapeutic outcome rather than signal amplification. Liposomes and lipid nanoparticles demonstrate excellent performance in this regard due to their high biocompatibility, predictable degradation, and ability to encapsulate a wide range of therapeutic agents. Their success in clinically approved formulations highlights that moderate analytical sensitivity can be sufficient when coupled with robust biological performance and translational readiness.

Polymeric nanoparticles and dendrimers provide additional advantages through their high structural tunability and controlled release capabilities [15]. Their analytical performance is expressed through precise cargo loading, stimulus-responsive release, and targeting specificity rather than optical or magnetic signal strength. These properties enable improved selectivity and functional performance in therapeutic applications, although complex synthesis and potential cytotoxicity remain limiting factors.

Hybrid nanomaterials aim to bridge the gap between high analytical sensitivity and favorable biological performance by integrating organic and inorganic components within a single platform [104]. For example, inorganic cores can provide strong imaging or sensing signals [105], while organic shells improve biocompatibility, stability, and targeting capability [106].

Such systems frequently demonstrate balanced performance across diagnostics, imaging, and therapy, making them particularly attractive for theranostic applications. Nevertheless, increased structural complexity may negatively affect reproducibility, scalability, and regulatory feasibility, highlighting an important trade-off between analytical performance and translational practicality. When comparing analytical performance across the entire application spectrum, it becomes evident that no single class of nanomaterials is universally superior. Inorganic nanomaterials dominate applications requiring maximal sensitivity and signal amplification, such as diagnostics and imaging. Organic nanocarriers excel in therapeutic applications where biological compatibility and controlled delivery are paramount. Hybrid systems offer the most comprehensive performance profile but at the cost of increased complexity. Therefore, optimal nanomaterial selection should be application-driven rather than performance-driven in a narrow analytical sense.

Overall, this comparative analysis underscores that analytical performance in nanobiotechnology must be interpreted within the context of intended biomedical use. High sensitivity and signal strength alone do not guarantee clinical success, while systems with moderate analytical characteristics may achieve superior outcomes through enhanced safety, reproducibility, and translational compatibility. Future developments in nanobiotechnology should therefore focus on balanced design strategies that integrate analytical excellence with biological relevance and regulatory feasibility.

## 9. Future Perspectives

Nanobiotechnology is rapidly advancing toward personalized medicine, which aims to tailor therapeutic and diagnostic strategies to individual patient characteristics, including genetic profiles, biomarkers, and treatment response. In this context, theranostic systems play a key role by integrating diagnostic and therapeutic functions within a single nanosystem, enabling real-time monitoring of treatment efficacy and adaptive therapeutic decision-making. Such approaches support more precise interventions, reduce adverse effects, and improve overall treatment outcomes, making them highly attractive for future clinical applications. Another important direction involves the development of intelligent and responsive nanobiological systems capable of reacting to specific biological signals or external stimuli [107]. These smart nanomaterials can be engineered to alter their behavior in response to changes in the disease microenvironment, such as pH, enzyme activity, temperature, or the presence of specific biomarkers [108]. This functionality enables spatially and temporally controlled drug release, enhancing therapeutic selectivity while minimizing off-target effects. Ongoing research suggests that such systems may offer significant advantages in the treatment of cancer, neurological disorders, and chronic inflammatory diseases. From a translational perspective, the successful implementation of nanobiotechnology in clinical practice depends on the ability to bridge the gap between laboratory-scale innovation and real-world medical application. Clinical translation has been most successful for relatively simple and well-characterized nanoplatforms, such as liposomes and lipid nanoparticles, due to their reproducible physicochemical properties, scalable manufacturing processes, and predictable biological behavior [109]. Notably, the clinical success of these platforms highlights the importance of simplified design and regulatory compatibility, which have enabled the approval of multiple lipid-based nanomedicines for therapeutic and diagnostic use. In contrast, many advanced nanobiotechnology concepts, including multifunctional theranostic systems and highly engineered nanobiosensors, remain largely confined to preclinical research due to challenges related to long-term safety evaluation, manufacturing complexity, and regulatory approval [110]. Bridging this translational gap requires the early integration of regulatory and clinical considerations into nanomaterial design and development. Standardized characterization methods, regulatory-aware study design, and the use of clinically relevant disease models are essential to improve reproducibility and comparability across studies. Furthermore, close interdisciplinary collaboration between material scientists, clinicians, toxicologists, and regulatory authorities will be critical for accelerating the safe and effective translation of nanobiotechnology from bench to bedside [111,112,113,114].

## 10. Conclusions

Nanobiotechnology encompasses a broad spectrum of applications that are already exerting a tangible impact on modern medicine, ranging from advanced drug delivery systems and molecular diagnostics to gene therapies and immunotherapies. Substantial progress has been achieved in the development of nanoscale drug carriers that improve bioavailability, enable targeted delivery, and reduce systemic toxicity, as well as in nanodiagnostic platforms that enhance biomarker detection sensitivity and support earlier disease diagnosis. Advances in nanostructured scaffolds have further strengthened tissue engineering and regenerative medicine by more closely mimicking the native extracellular matrix. These achievements are particularly evident in oncology, neurology, and infectious diseases, where nanotechnology-based strategies increasingly complement or outperform conventional approaches. A key lesson emerging from these developments is that the most successful clinical implementations of nanobiotechnology have been based on relatively simple and well-characterized nanoplatforms, such as liposomes and lipid nanoparticles. These systems have demonstrated clear translational potential by effectively addressing specific limitations of traditional therapies, including poor solubility, limited stability, and insufficient targeting. In contrast, more complex multifunctional nanosystems, while scientifically promising, largely remain at the experimental stage. Despite the significant progress achieved to date, several challenges remain unresolved and continue to limit the broader clinical adoption of nanobiotechnology. These challenges include an incomplete understanding of the long-term toxicity and immunological effects, variability in nanomaterial physicochemical properties, difficulties in achieving scalable and reproducible manufacturing, and the lack of harmonized regulatory and toxicological assessment frameworks. Future efforts in nanobiotechnology should therefore prioritize translationally oriented research focused on standardized material characterization, comprehensive safety evaluation, and manufacturing scalability. Early consideration of regulatory requirements, together with interdisciplinary collaboration between material scientists, clinicians, and regulatory experts, will be essential to ensure that continued advances in nanobiotechnology translate into safe, effective, and clinically meaningful medical applications.

## Figures and Tables

**Table 1:** Overview of major nanomaterial classes used in nanobiotechnology, highlighting their key advantages, analytical and therapeutic performance, main limitations, and representative biomedical applications.

Type of Nanomaterial	Key Advantages	Analytical/Therapeutic Performance	Main Limitations	Typical Applications	References
**Liposomes & lipid nanoparticles**	High biocompatibility, clinical approval, efficient encapsulation of drugs and nucleic acids	Good delivery efficiency; moderate analytical sensitivity; excellent translational potential	Limited structural stability, possible premature drug leakage	Drug delivery, gene therapy, vaccines	[3,8]
**Polymeric nanoparticles & dendrimers**	High structural tunability, controlled release, easy functionalization	High targeting specificity and controlled release; good analytical selectivity	Potential cytotoxicity depending on polymer type; complex synthesis	Targeted therapy, gene delivery, theranostics	[10]
**Metallic nanoparticles (e.g., Au, Fe_3_O_4_)**	Unique optical/magnetic properties, signal amplification	Very high analytical sensitivity and imaging performance	Limited biodegradability, long-term accumulation, toxicity concerns	Biosensors, imaging, diagnostics	[11]
**Semiconducting nanoparticles (quantum dots)**	Strong fluorescence, photostability, tunable emission	Excellent sensitivity for bioimaging and biomarker detection	Toxicity of heavy metals, limited clinical translation	Fluorescence imaging, molecular diagnostics	[12]
**Hybrid nanomaterials**	Combination of organic and inorganic advantages	Balanced high analytical performance and therapeutic efficiency	More complex design and characterization	Theranostics, multimodal imaging, precision medicine	[12,13]

## Data Availability

No new data were created or analyzed in this study. Data sharing is not applicable to this article.

## References

[1] Jain K.K. (2008). Nanomedicine: Application of nanobiotechnology in medical practice. Med. Princ. Pract..

[2] Chattopadhyay S., Goswami A., Sil M. (2025). Nanobiotechnology: Traditional re-interpreting personalized medicine through targeted therapies and regenerative solutions. Naunyn Schmiedebergs Arch. Pharmacol..

[3] Jia Y., Jiang Y., He Y., Zhang W., Zou J., Magar K.T., Boucetta H., Teng C., He W. (2023). Approved Nanomedicine against Diseases. Pharmaceutics.

[4] Durgam L.K., Oroszi T.L. (2025). Revolutionizing healthcare: The transformative potential of nanotechnology in medicine. Front. Drug Deliv..

[5] Forgham H., Chang Y., Wang Y., Zhu J., Liu L., Biggs H., Kakinen A., Jiang Y., You X., Thurecht K.J. (2025). The evolution of nanomedicine: The rise of next-generation nanomaterials in cancer nanomedicine. Sci. Adv..

[6] Jain K.K. (2011). Nanobiotechnology and personalized medicine. Prog. Mol. Biol. Transl. Sci..

[7] Malik S., Muhammad K., Waheed Y. (2023). Emerging Applications of Nanotechnology in Healthcare and Medicine. Molecules.

[8] Ashraf M., Zulfiqar F., Ijaz U., Rauf U., Riaz M., Arshad S., Jamil Khan M., Sahin T. (2025). Nanotechnology, nano-systems and applications of nanoparticles in novel drug delivery—A comprehensive review. Pak. J. Pharm. Sci..

[9] Rahman M.A., Jalouli M., Yadab M.K., Al-Zharani M. (2025). Progress in Drug Delivery Systems Based on Nanoparticles for Improved Glioblastoma Therapy: Addressing Challenges and Investigating Opportunities. Cancers.

[10] Wakaskar R.R. (2018). General overview of lipid-polymer hybrid nanoparticles, dendrimers, micelles, liposomes, spongosomes and cubosomes. J. Drug Target..

[11] Sun L., Liu H., Ye Y., Lei Y., Islam R., Tan S., Tong R., Miao Y.B., Cai L. (2023). Smart nanoparticles for cancer therapy. Signal Transduct. Target. Ther..

[12] Sivadasan D., Sultan M.H., Madkhali O., Almoshari Y., Thangavel N. (2021). Polymeric Lipid Hybrid Nanoparticles (PLNs) as Emerging Drug Delivery Platform—A Comprehensive Review of Their Properties, Preparation Methods, and Therapeutic Applications. Pharmaceutics.

[13] Jacob S., Varkey N.R., Boddu S.H.S., Gorain B., Rao R., Nair A.B. (2025). Advances in Lipid-Polymer Hybrid Nanoparticles: Design Strategies, Functionalization, Oncological and Non-Oncological Clinical Prospects. Pharmaceuticals.

[14] Mendonça M.C.P., Kont A., Kowalski P.S., O’Driscoll C.M. (2023). Design of lipid-based nanoparticles for delivery of therapeutic nucleic acids. Drug Discov. Today..

[15] Eltaib L. (2025). Polymeric Nanoparticles in Targeted Drug Delivery: Unveiling the Impact of Polymer Characterization and Fabrication. Polymers.

[16] Saker R., Regdon G., Sovány T. (2024). Pharmacokinetics and toxicity of inorganic nano-particles and the physicochemical properties/factors affecting them. J. Drug Deliv. Sci. Technol..

[17] Diez-Pascual A.M., Rahdar A. (2022). Functional Nanomaterials in Biomedicine: Current Uses and Potential Applications. ChemMedChem.

[18] Panico S., Capolla S., Bozzer S., Toffoli G., Dal B.M., Macor P. (2022). Biological Features of Nanoparticles: Protein Corona Formation and Interaction with the Immune System. Pharmaceutics.

[19] Salvati A., Pitek A.S., Monopoli M.P., Prapainop K., Bombelli F.B., Hristov D.R., Kelly P.M., Åberg C., Mahon E., Dawson K.A. (2013). Transferrin-functionalized nanoparticles lose their targeting capabilities when a biomolecule corona adsorbs on the surface. Nat. Nano-Technol..

[20] Díez-Pascual A.M. (2022). Surface Engineering of Nanomaterials with Polymers, Biomolecules, and Small Ligands for Nanomedicine. Materials.

[21] Kyriakides T.R., Raj A., Tseng T.H., Xiao H., Nguyen R., Mohammed F.S., Halder S., Xu M., Wu M.J., Bao S. (2021). Biocompatibility of nanomaterials and their immunological properties. Biomed. Mater..

[22] Souri M., Soltani M., Moradi Kashkooli F., Kiani Shahvandi M., Chiani M., Shariati F.S., Mehrabi M.R., Munn L.L. (2022). Towards principled design of cancer nanomedicine to accelerate clinical translation. Mater. Today Bio.

[23] Hirsjärvi S., Passirani C., Benoit J.P. (2011). Passive and active tumour targeting with nanocarriers. Curr. Drug Discov. Technol..

[24] Attia M.F., Anton N., Wallyn J., Omran Z., Vandamme T.F. (2019). An overview of active and passive targeting strategies to improve the nanocarriers efficiency to tumour sites. J. Pharm. Pharmacol..

[25] Biffi S., Voltan R., Bortot B., Zauli G., Secchiero P. (2019). Actively targeted nanocarriers for drug delivery to cancer cells. Expert Opin. Drug Deliv..

[26] Nogueira-Librelotto D.R., Codevilla C.F., Farooqi A., Rolim C.M. (2017). Transferrin-Conjugated Nanocarriers as Active-Targeted Drug Delivery Platforms for Cancer Therapy. Curr. Pharm. Des..

[27] Wu J. (2021). The Enhanced Permeability and Retention (EPR) Effect: The Significance of the Concept and Methods to Enhance Its Application. J. Pers. Med..

[28] Subhan M.A., Yalamarty S.S.K., Filipczak N., Parveen F., Torchilin V.P. (2021). Recent Advances in Tumor Targeting via EPR Effect for Cancer Treatment. J. Pers. Med..

[29] Subhan M.A., Parveen F., Filipczak N., Yalamarty S.S.K., Torchilin V.P. (2023). Approaches to Improve EPR-Based Drug Delivery for Cancer Therapy and Diagnosis. J. Pers. Med..

[30] Zhang J., Lin Y., Lin Z., Wei Q., Qian J., Ruan R., Jiang X., Hou L., Song J., Ding J. (2022). Stimuli-Responsive Nanoparticles for Controlled Drug Delivery in Synergistic Cancer Immunotherapy. Adv. Sci..

[31] Lee J.H., Yeo Y. (2015). Controlled Drug Release from Pharmaceutical Nanocarriers. Chem. Eng. Sci..

[32] Wang Y., Huang R., Feng S., Mo R. (2025). Advances in nanocarriers for targeted drug delivery and controlled drug release. Chin. J. Nat. Med..

[33] Zhang X., Zhang H., Liu X., Wang J., Li S., Gao P. (2024). Review and Future Perspectives of Stimuli-Responsive Bridged Polysilsesquioxanes in Controlled Release Applications. Polymers.

[34] Islam S., Ahmed M.M.S., Islam M.A., Hossain N., Chowdhury M.A. (2025). Advances in nanoparticles in targeted drug delivery—A review. Results Surf. Interfaces.

[35] Antonelli A., Palma F. (2025). Nanocarrier-Based Delivery Systems for Natural Compounds Across Research Stages. Materials.

[36] Parvin N., Aslam M., Alam M.N., Mandal T.K. (2025). Nanotechnology Driven Innovations in Modern Pharmaceutics: Therapeutics, Imaging, and Regeneration. Nanomaterials.

[37] Jain K.K. (2020). Role of Nanobiotechnology in Drug Delivery. Methods Mol. Biol..

[38] Williams A.D., So A., Tchou J. (2018). Overall survival is similar between women who seek care at one or more institutions after diagnosis of operable breast cancer in the community. Surg. Oncol..

[39] Yu S.J., Peng W.J., Zhang H., Chen X.Z., Wei M.H., Yan W.R. (2019). Association between both maternal and fetal angiotensinogen gene single nucleotide polymorphism and preeclampsia/eclampsia. Zhonghua Liu Xing Bing Xue Za Zhi.

[40] Joshi D.C., Prasad S., Bhati V., Sharma P.K., Joshi N., Durgapal S., Chavan M.B., Maurya V.K., Subramaniyan V., Paudel K.R. (2026). Revolutionizing cancer treatment: Nanotherapeutics targeting the tumor micro-environment. Colloids Surf. B Biointerfaces.

[41] Saraswathi T.S., Mothilal M., Bukke S.P.N., Thalluri C., Chettupalli A.K. (2025). Recent advances in potential drug nanocarriers for CNS disorders: A review. Biomed. Eng. Online.

[42] Seymour J.F. (2020). Venetoclax plus obinutuzumab therapy for front-line treatment of chronic lymphocytic leukaemia. Lancet Oncol..

[43] Gieroń Ż., Sitko K., Zieleźnik-Rusinowska P., Szopiński M., Rojek-Jelonek M., Rostański A., Rudnicka M., Małkowski E. (2021). Ecophysiology of Arabidopsis arenosa, a new hyperaccumulator of Cd and Zn. J. Hazard. Mater..

[44] Oliveira B.B., Ferreira D., Fernandes A.R., Baptista P.V. (2023). Engineering gold nanoparticles for molecular diagnostics and biosensing. Wiley Interdiscip. Rev. Nanomed. Nanobiotechnol..

[45] Chamorro-Garcia A., Merkoçi A. (2016). Nanobiosensors in diagnostics. Nanobiomedicine.

[46] Jain K.K. (2007). Applications of nanobiotechnology in clinical diagnostics. Clin. Chem..

[47] Nie S. (2009). Biomedical nanotechnology for molecular imaging, diagnostics, and targeted therapy. Annu. Int. Conf. IEEE Eng. Med. Biol. Soc..

[48] Jain K.K. (2003). Nanodiagnostics: Application of nanotechnology in molecular diagnostics. Expert Rev. Mol. Diagn..

[49] Bardhan N. (2022). Nanomaterials in diagnostics, imaging and delivery: Applications from COVID-19 to cancer. MRS Commun..

[50] Kim C., Kang M.S., Raja I.S., Joung Y.K., Han D.W. (2024). Advancements in nanobiosensor technologies for in-vitro diagnostics to point of care testing. Heliyon.

[51] Iyer K.A., Tenchov R., Ralhan K., Birg R.E., Lotti Diaz L.M., Hughes K.J., Ganesan M., Ivanov J.M., Zhou Q.A. (2025). Nanoscale Materials in Biomedical Applications of Sensors: Insights from a Comprehensive Landscape Analysis. ACS Appl. Nano Mater..

[52] Khalid A., Tomljenovic-Hanic S. (2024). Emerging Fluorescent Nanoparticles for Non-Invasive Bioimaging. Molecules.

[53] Han X., Xu K., Taratula O., Farsad K. (2019). Applications of nanoparticles in biomedical imaging. Nanoscale.

[54] Elendu C., Amaechi D.C., Elendu T.C., Amaechi E.C., Elendu I.D., Omeludike J.C., Omeludike E.K., Onubogu N.C., Ogelle E.C., Meduoye O.O.M. (2025). Essential information about nanotechnology in cardiology. Ann. Med. Surg..

[55] Hsu J.C., Tang Z., Eremina O.E., Sofias A.M., Lammers T., Lovell J.F., Zavaleta C., Cai W., Cormode D.P. (2023). Nanomaterial-based contrast agents. Nat. Rev. Methods Primers.

[56] Key J., Leary J.F. (2014). Nanoparticles for multimodal in vivo imaging in nanomedicine. Int. J. Nanomed..

[57] Lee S., Rafiq S., Kang S.H. (2025). Nanobiosensors for Single-Molecule Diagnostics: Toward Integration with Super-Resolution Imaging. Biosensors.

[58] Esporrín-Ubieto D., Fraire J.C., Sánchez-deAlcázar D., Sánchez S. (2025). Engineered Plasmonic and Fluorescent Nanomaterials for Biosensing, Motion, Imaging, and Therapeutic Applications. Adv. Mater..

[59] Ramoso J.P., Rasekh M., Balachandran W. (2025). Graphene-Based Biosensors: Enabling the Next Generation of Diagnostic Technologies-A Review. Biosensors.

[60] Szunerits S., Rodrigues T., Bagale R., Happy H., Boukherroub R., Knoll W. (2024). Graphene-based field-effect transistors for biosensing: Where is the field heading to?. Anal. Bioanal. Chem..

[61] Wang M., Jin L., Hang-Mei Leung P., Wang-Ngai Chow F., Zhao X., Chen H., Pan W., Liu H., Li S. (2024). Advancements in magnetic nanoparticle-based biosensors for point-of-care testing. Front. Bioeng. Biotechnol..

[62] Loskutova A., Seitkali A., Aliyev D., Bukasov R. (2025). Quantum Dot-Based Luminescent Sensors: Review from Analytical Perspective. Int. J. Mol. Sci..

[63] Smith I.O., Liu X.H., Smith L.A., Ma P.X. (2009). Nanostructured polymer scaffolds for tissue engineering and regenerative medicine. Wiley Interdiscip. Rev. Nanomed. Nanobiotechnol..

[64] Smith L.A., Ma P.X. (2004). Nano-fibrous scaffolds for tissue engineering. Colloids Surf. B Biointerfaces.

[65] Pisani S., Evangelista A., Chesi L., Croce S., Avanzini M.A., Dorati R., Genta I., Benazzo M., Comoli P., Conti B. (2025). Nanofibrous Scaffolds’ Ability to Induce Mesenchymal Stem Cell Differentiation for Soft Tissue Regenerative Applications. Pharmaceuticals.

[66] Kim C.D., Koo K.M., Kim H.J., Kim T.H. (2024). Recent Advances in Nanomaterials for Modulation of Stem Cell Differentiation and Its Therapeutic Applications. Biosensors.

[67] Polo Y., Luzuriaga J., Iturri J., Irastorza I., Toca-Herrera J.L., Ibarretxe G., Unda F., Sarasua J.R., Pineda J.R., Larrañaga A. (2021). Nanostructured scaffolds based on bioresorbable polymers and graphene oxide induce the aligned migration and accelerate the neuronal differentiation of neural stem cells. Nanomedicine.

[68] Echeverria Molina M.I., Malollari K.G., Komvopoulos K. (2021). Design Challenges in Polymeric Scaffolds for Tissue Engineering. Front. Bioeng. Biotechnol..

[69] Zhao X., Li N., Zhang Z., Hong J., Zhang X., Hao Y., Wang J., Xie Q., Zhang Y., Li H. (2024). Beyond hype: Unveiling the Real challenges in clinical translation of 3D printed bone scaffolds and the fresh prospects of bioprinted organoids. J. Nanobiotechnol..

[70] Farjaminejad S., Farjaminejad R., Garcia-Godoy F. (2024). Nanoparticles in Bone Regeneration: A Narrative Review of Current Advances and Future Directions in Tissue Engineering. J. Funct. Biomater..

[71] Sangnim T., Puri V., Dheer D., Venkatesh D.N., Huanbutta K., Sharma A. (2024). Nanomaterials in the Wound Healing Process: New Insights and Advancements. Pharmaceutics.

[72] Chaudhury K., Kumar V., Kandasamy J., RoyChoudhury S. (2014). Regenerative nanomedicine: Current perspectives and future directions. Int. J. Nanomed..

[73] Kubinová S., Syková E. (2010). Nanotechnologies in regenerative medicine. Minim. Invasive Ther. Allied Technol..

[74] Xu H., Li Z., Si J. (2014). Nanocarriers in gene therapy: A review. J. Biomed. Nanotechnol..

[75] Mollé L.M., Smyth C.H., Yuen D., Johnston A.P.R. (2022). Nanoparticles for vaccine and gene therapy: Overcoming the barriers to nucleic acid delivery. Wiley Interdiscip. Rev. Nanomed. Nanobiotechnol..

[76] Gómez-Aguado I., Rodríguez-Castejón J., Vicente-Pascual M., Rodríguez-Gascón A., Solinís M.Á., Del Pozo-Rodríguez A. (2020). Nanomedicines to Deliver mRNA: State of the Art and Future Perspectives. Nanomaterials.

[77] Hamimed S., Jabberi M., Chatti A. (2022). Nanotechnology in drug and gene delivery. Naunyn Schmiedebergs Arch. Pharmacol..

[78] Zhang Y., Luo J., Gui X., Zheng Y., Schaar E., Liu G., Shi J. (2023). Bioengineered nanotechnology for nucleic acid delivery. J. Control. Release.

[79] Desai N., Chavda V., Singh T.R.R., Thorat N.D., Vora L.K. (2024). Cancer Nanovaccines: Nanomaterials and Clinical Perspectives. Small.

[80] Raziq K., Xue T., Sun D. (2025). The shift toward nanovaccination: A comprehensive review of advancing nanovaccination for combinatory immune regulation therapies to treat infectious diseases and cancer. Int. Immunopharmacol..

[81] Grimaudo M.A. (2021). Nanotechnology for the Development of Nanovaccines in Cancer Immunotherapy. Adv. Exp. Med. Biol..

[82] Yu M., Yang W., Yue W., Chen Y. (2022). Targeted Cancer Immunotherapy: Nanoformulation Engineering and Clinical Translation. Adv. Sci..

[83] Wells K., Liu T., Zhu L., Yang L. (2024). Immunomodulatory nanoparticles activate cytotoxic T cells for enhancement of the effect of cancer immunotherapy. Nanoscale.

[84] Feng X., Xu W., Li Z., Song W., Ding J., Chen X. (2019). Immunomodulatory Nanosystems. Adv. Sci..

[85] Kim M., Shin M., Zhao Y., Ghosh M., Son Y.O. (2024). Transformative Impact of Nanocarrier-Mediated Drug Delivery: Overcoming Biological Barriers and Expanding Therapeutic Horizons. Small Sci..

[86] Zelepukin I.V., Shevchenko K.G., Deyev S.M. (2024). Rediscovery of mononuclear phagocyte system blockade for nanoparticle drug delivery. Nat. Commun..

[87] Berikkhanova K., Inuwa I., Jibo A.G., Berikkhanov N., Bikhanov N., Sultan Y., Omarbekov A. (2026). Hybrid Nanocarriers for Cancer Therapy: Advancements in Co-Delivery of Gene Therapy and Immunotherapy. Int. J. Mol. Sci..

[88] Akçan R., Aydogan H.C., Yildirim M.Ş., Taştekin B., Sağlam N. (2020). Nanotoxicity: A challenge for future medicine. Turk. J. Med. Sci..

[89] Bondarenko O., Mortimer M., Kahru A., Feliu N., Javed I., Kakinen A., Lin S., Xia T., Song Y., Davis T.P. (2021). Nanotoxicology and Nanomedicine: The Yin and Yang of Nano-Bio Interactions for the New Decade. Nano Today.

[90] Hannon G., Lysaght J., Liptrott N.J., Prina-Mello A. (2019). Immunotoxicity Considerations for Next Generation Cancer Nanomedicines. Adv. Sci..

[91] Aljabali A.A., Obeid M.A., Bashatwah R.M., Serrano-Aroca Á., Mishra V., Mishra Y., El-Tanani M., Hromić-Jahjefendić A., Kapoor D.N., Goyal R. (2023). Nanomaterials and Their Impact on the Immune System. Int. J. Mol. Sci..

[92] Mangla B., Kumar P., Javed S., Pathan T., Ahsan W., Aggarwal G. (2025). Regulating nanomedicines: Challenges, opportunities, and the path forward. Nanomedicine.

[93] Csóka I., Ismail R., Jójárt-Laczkovich O., Pallagi E. (2021). Regulatory Considerations, Challenges and Risk-based Approach in Nanomedicine Development. Curr. Med. Chem..

[94] Bi J., Mo C., Li S., Huang M., Lin Y., Yuan P., Liu Z., Jia B., Xu S. (2023). Immunotoxicity of metal and metal oxide nanoparticles: From toxic mechanisms to metabolism and outcomes. Biomater. Sci..

[95] Mehmood S., Iraqui S., Ojha R.K., Sharma N., Marlinda A.R. (2025). Therapeutic potential and toxicological challenges of metal nanoparticles in drug delivery: A comprehensive review. Nanomedicine.

[96] Mondal J., An J.M., Surwase S.S., Chakraborty K., Sutradhar S.C., Hwang J., Lee J., Lee Y.-K. (2022). Carbon Nanotube and Its Derived Nanomaterials Based High Performance Biosensing Platform. Biosensors.

[97] Herdiana Y. (2025). Bridging the Gap: The Role of Advanced Formulation Strategies in the Clinical Translation of Nanoparticle-Based Drug Delivery Systems. Int. J. Nanomed..

[98] Hang Y., Wang A., Wu N. (2024). Plasmonic silver and gold nanoparticles: Shape- and structure-modulated plasmonic functionality for point-of-caring sensing, bio-imaging and medical therapy. Chem. Soc. Rev..

[99] Kiani-Salmi N., Bahramian B., Abedi-Firoozjah R., Ebrahimi A., Khezerlou A., Tavassoli M., Ehsani A. (2025). Recent advances and trends in magnetic nanoparticle-assisted aptasensors for foodborne bacteria monitoring: Applications, challenges, and updates. Food Chem. X.

[100] Navid P., Akbari-Hasanjani H.R., Akbari-Hasanjani R. (2025). A Comprehensive Study of Quantum Dots: Ranging From Synthesis to Applications in Electrochemical Biosensors in the Detection of Biomolecules, Gastrointestinal Diseases, and Electrophysiology. Nano Sel..

[101] Yücer S., Sarac B., Ciftci F. (2025). Tissue engineering and biosensing applications of carbon-based nanomaterials. Biomed. Eng. Adv..

[102] Ravi S.N., Rajendran S., Madhumathi G.S., Packirisamy A.S.B., Vallinayagam S., Khan A.A., Malik A. (2024). Carbon nanomaterials: Pioneering innovations in bioimaging and biosensing technologies. J. Mol. Struct..

[103] Yu L., Zhu Y., Zheng X., Huang R., Song S., Liu Y., Liu Z., Chen B., Zhu R. (2025). Material-based neuroimaging and biomarker detection for central nervous system disorder. Mater. Today Bio.

[104] Park W., Shin H., Choi B., Rhim W.K., Na K., Keun Han D. (2020). Advanced Hybrid Nanomaterials for Biomedical Applications. Prog. Mater. Sci..

[105] Du W., Zhou L., Zhang Q., Liu X., Wei X., Li Y. (2021). Inorganic Nanomaterial for Biomedical Imaging of Brain Diseases. Molecules.

[106] Yanar F., Carugo D., Zhang X. (2023). Hybrid Nanoplatforms Comprising Organic Nanocompartments Encapsulating Inorganic Nanoparticles for Enhanced Drug Delivery and Bioimaging Applications. Molecules.

[107] Singh N., Kaur A., Madhu A., Yadav M. (2025). Advancements in nanotechnology for biomedical and wearable applications. Next Mater..

[108] Chen X., Wu D., Chen Z. (2024). Biomedical applications of stimuli-responsive nanomaterials. MedComm.

[109] Desai N., Rana D., Patel M., Bajwa N., Prasad R., Vora L.K. (2025). Nanoparticle Therapeutics in Clinical Perspective: Classification, Marketed Products, and Regulatory Landscape. Small.

[110] He X., Wang L., Zhang T., Lu T. (2025). Progress in the Application of Nanomaterials in Tumor Treatment. Biomed..

[111] Premcheska S., Skirtach A.G., Kaczmarek A.M. (2025). Roadmap to Nanomedical Applications: Nanotoxicology and In Vitro Guidelines for Lanthanide-Doped Luminescence Nanothermometers. Adv. Nanobiomed. Res..

[112] Huang W., Zhou J., Liang Y., Saladin R.J., Li L., Kang L., Hua C., Cai W. (2025). Application of smart responsive nanomaterials in the theranostics of gastrointestinal malignancies: Current status and future perspectives. Coord. Chem. Rev..

[113] Dai X.J., Li W.J., Xie D.D., Liu B.X., Gong L., Han H.H. (2025). Stimuli-Responsive Nano Drug Delivery Systems for the Treatment of Neurological Diseases. Small.

[114] Rehan F., Zhang M., Fang J., Greish K. (2024). Therapeutic Applications of Nanomedicine: Recent Developments and Future Perspectives. Molecules.

